# Novel Human Bufavirus Genotype 3 in Children with Severe Diarrhea, Bhutan

**DOI:** 10.3201/eid2006.131430

**Published:** 2014-06

**Authors:** Takaaki Yahiro, Sonam Wangchuk, Kinlay Tshering, Purushotam Bandhari, Sangay Zangmo, Tshering Dorji, Karchung Tshering, Takashi Matsumoto, Akira Nishizono, Maria Söderlund-Venermo, Kamruddin Ahmed

**Affiliations:** Faculty of Medicine, Oita University, Yufu, Japan (T. Yahiro, T. Matsumoto, A. Nishizono, K. Ahmed);; Ministry of Health, Thimphu, Bhutan (S. Wangchuk, S. Zangmo, T. Dorji, K. Tshering);; Jigme Dorji Wangchuk National Referral Hospital, Thimphu (K. Tshering);; Mongar Regional Referral Hospital, Mongar, Bhutan (P. Bandhari);; University of Helsinki, Helsinki, Finland (M. Söderlund-Venermo)

## Abstract

We identified a new genotype of bufavirus, BuV3, in fecal samples (0.8%) collected to determine the etiology of diarrhea in children in Bhutan. Norovirus GII.6 was detected in 1 sample; no other viral diarrheal pathogens were detected, suggesting BuV3 as a cause of diarrhea. This study investigates genetic diversity of circulating BuVs.

In 2012, a novel parvovirus, bufavirus (BuV), was discovered in fecal samples of children with diarrhea in Burkina Faso (*1*). The virus belongs to the species primate protoparvovirus 1 of the genus *Protoparvovirus* (*2*). BuV has a single-stranded DNA genome and encodes nonstructural protein 1 (NS1) and viral structural proteins 1 and 2 (VP1 and VP2). Two genotypes, BuV1 and BuV2, have been described; the highly diverse capsid gene indicates the possibility of further genotypes of this virus (*1*).

One research group, which used PCR to test fecal samples collected in 3 countries, had previously found various proportions of specimens positive for BuV: 4 of 98 (4%) in Burkina Faso, 1 of 63 (1.6%) in Tunisia, and none of 100 in Chile (*1*). Fecal samples from Tunisia were from children with acute flaccid paralysis; samples from Burkina Faso and Chile were from children with diarrhea. It is not known whether BuV is pathogenic in humans.

Bhutan is a small landlocked country between India and China; an estimated 23% of the people of Bhutan live below the poverty line (*3*). The population is mainly concentrated in the capital, Thimphu (altitude 2,248–2,648 m), and is otherwise sparsely distributed throughout the country. Diarrhea is a major cause of illness and death among children in Bhutan. Irrespective of severity, hospitalization, or causative agents, the annual morbidity rate for children <5 years of age with diarrhea is 168.8/1,000 (17%) (*4*); however, the etiology of diarrhea in this country has not been studied in detail. We conducted this study to investigate the genetic diversity of circulating BuVs and to clarify the public health significance of BuV in Bhutan.

## The Study

As part of a project to identify viral etiology of diarrhea, 393 fecal samples were collected from children <5 years of age with watery diarrhea attending 2 hospitals in Bhutan. During February 2010–January 2012, 381 fecal samples (109 in 2010, 185 in 2011, and 87 in 2012) were collected from hospitalized patients or outpatients of Jigme Dorji Wangchuk National Referral Hospital. This hospital mainly serves the population of Thimphu and is the only national reference hospital in the country. In April 2010, 12 more samples were obtained from children hospitalized in Mongar Regional Referral Hospital. This hospital mainly serves the population of Mongar and is the reference hospital for the eastern part of the country. The Research Ethics Board of Health in Bhutan approved this study.

Viral genomic DNA was extracted from fecal samples by using the QIAamp Viral RNA Mini Kit (QIAGEN Sciences, Valencia, CA, USA) according to the manufacturer’s instructions. The presence of BuV was determined by nested PCR targeting the NS1 region (*1*). For whole-genome sequencing of BuV3, primers were constructed from consensus regions after aligning the whole-genome sequences of BuV1. BuV-positive samples were tested for norovirus, bocavirus, adenovirus, astrovirus, salivirus, cosavirus, and aichivirus by PCR, and for rotavirus by enzyme immunoassay (*5*–*8*). No tests for diarrheic bacteria were done on these samples. PCR amplicons were directly sequenced to confirm the findings and to compare the sequences. Nucleotide sequencing was performed by using the BigDye Terminator v3.1 Cycle Sequencing kit (Applied Biosystems, Foster City, CA USA) according to the manufacturer’s instructions. Multiple sequence alignment was done by using MUSCLE (*9*) and the phylogenetic tree was constructed by using the neighbor-joining method by MEGA 5 (*10*). A bootstrap analysis of 1,000 replicates was done to find the significance of branching.

Three of 393 samples (0.8%) were positive for BuV DNA. The overall proportion of positive samples of BuV in Thimphu was 0.5% (2 samples); 1 (0.9%) was detected in 2010 and the other (1.1%) in 2011. No samples from Thimphu were BuV-positive in 2012. In addition, 1 sample (8.3%) from Mongar was BuV-positive. The demographic data of these patients are shown in Table 1. In 1 of the 3 BuV-positive fecal samples, norovirus GII.6 was also detected; in the other 2 samples, the enteric viruses tested for were not detected.

**Table 1:** Demographic information and tested diarrheagenic viruses in the stool samples of hospital inpatients with bufavirus-positive diarrhea, Bhutan, 2010–2011*

ID no.	Age, mo/ sex	Underlying condition	Sample collection date	Hospital	Other viruses tested for							
					RV	ADV	BCV	ASV	NRV	SLV	CSV	ACV
BTN-63	18/M	None	2010 Apr 26	MRR	−	−	−	−	−	−	−	−
BTN-109	1/M	None	2010 Nov 2	JDWNR	−	−	−	−	−	−	−	−
BTN-310	31/F	Ventricular septal defect	2011 Dec 12	JDWNR	−	−	−	−	G II.6	−	−	−

*ID, identification; RV, rotavirus; ADV, adenovirus; BCV, bocavirus; ASV, astrovirus; NRV, norovirus; SLV, salivirus; CSV, cosavirus; ACV, aichivirus; −, negative; MRR, Mongar Regional Referral Hospital; JDWNR, Jigme Dorji Wangchuk National Referral Hospital.

The near-complete genome sequences of the 3 BuVs collected in Bhutan were obtained. The lengths of the open reading frames of NS1, VP1, and VP2 of the Bhutanese BuV genes were different from those of BuV1 and BuV2 (Table 2). Phylogenetic analyses, with a bootstrap value of 100, of the deduced amino acid sequence of NS1, VP1 (Figure), and VP2 genes showed that the Bhutanese BuVs consistently formed a cluster different from the genotypes BuV1 and BuV2. The NS1, VP1, and VP2 of the Bhutanese isolates showed ≈99% nucleotide and amino acid identities among themselves. Compared with BuV1, the respective NS1, VP1, and VP2 nt (amino acid) identities of the Bhutanese BuVs were 96% (95%–96%), 83% (77%–78%), and 79%–80% (73%), and compared with BuV2, 96% (95%), 80% (71%), and 76% (65%). Phylogenetic analysis and identity data support the finding of a new genotype, BuV3, in Bhutan. Similar to BuV1 and BuV2, all isolates collected in Bhutan contained an ATP- or GTP-binding Walker loop, phospholipase A_2_, 2 conserved replication initiator motifs, and a glycine-rich sequence; they also had the same splice sites. In the Bhutanese BuVs, 3 tandem repeats, TAGTTGATAAGT, TAGTTTATAAGT, and TAGTTTATAAAT, in the 3′ untranslated region (UTR) occurred at a frequency of 0, 6, or 3 times, and 0 or 1 times, respectively (Table 2). These results show that BuV3 3′UTR differs from 3′UTR of the other 2 genotypes.

**Table 2:** Comparison of the length of the nucleotide sequences of nearly complete genome, 5′ UTR, NS1, VP1, VP2, and 3′ UTR of strains of different bufavirus genotypes, Bhutan, 2010-2012*†

Strain	Genotype	Total	5′ UTR	NS1	VP1	VP2	3′ UTR	TAGTTGATAAGT	TAGTTTATAAGT	TAGTTTATAAAT
BTN-63	BuV3	4,733	ND	2,022	2,133	1,719	224	0	3	0
BTN-109	BuV3	4,734	ND	2,022	2,133	1,719	225	0	6	0
BTN-310	BuV3	4,766	ND	2,022	2,133	1,719	259	0	3	1
BF.96	BuV1	4,912	18	2,016	2,124	1,710	400	3	1	1
BF.86	BuV1	4,822	1	2,016	2,124	1,710	327	2	1	1
BF.7	BuV1	4,822	1	2,016	2,124	1,710	327	3	1	1
BF.39	BuV2	4,562	ND	2,022	2,124	1,710	62	ND	ND	ND

*NS, nucleotide sequence; UTR, untranslated region; VP, viral protein; ND, no data:sequence could not be determined. †Only partial sequence of 3′ UTR regions have been achieved in all strains. Nucleotide sequence of 5′ UTR of BuV strains from samples from Bhutan could not be determined.

**Figure:**
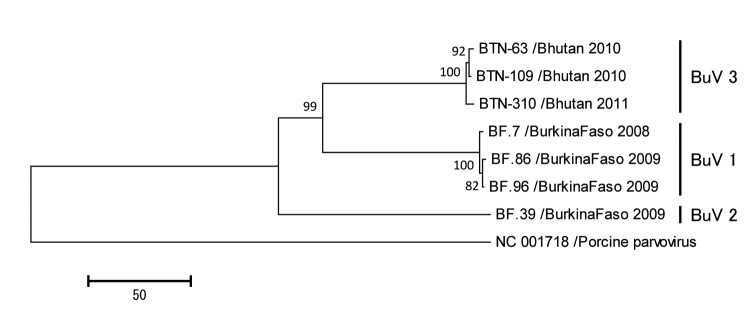
Phylogenetic trees of the viral protein 1 (VP1) of bufaviruses, constructed by using deduced amino acid sequences by neighbor-joining method. The full length open reading frames of VP1 genes were used to deduce amino acid sequences. Porcine parvovirus strain NC 001718 was used as an out-group. The numbers adjacent to the nodes represent the bootstrap values, and values <50% are not shown. Scale bar indicates the genetic distances expressed as amino acid substitutions per site. Accession numbers for DNA Data Bank of Japan, European Molecular Biology Laboratory, and GenBank are AB847987, AB847988, and AB847989 for strains BTN-63, BTN-109, and BTN-310, respectively.

## Conclusion

Genetic analyses of BuVs from Bhutan revealed a new genotype that may cause severe diarrhea in children. The absence of other diarrhea-causing viruses tested for in 2 of the 3 samples containing BuV3 supports a possible role for this parvovirus in diarrhea in infants; however, direct proof of causation is necessary (*11*,*12*). A more extensive testing for other known pathogens by using virochip microarrays, metagenomics, and direct testing for pathogenic bacteria will help determine whether BuVs alone can cause diarrhea.

Phylogenetic analyses and identity data indicate that BuV3 is a new genotype. The identity data between BuV3 and other genotypes are comparable to the data found by Phan et al., wherein genotypes BuV1 and BuV2 showed 95% identity in the NS1 region, but only 72% identity in the capsid region (*1*). In BuV1, the 3 tandem repeats each occurred with a frequency of 3 or 2 times, once, and once, respectively. This differs substantially from that of BuV3 found in Bhutan, further revealing the molecular characteristics of the new genotype.

In conclusion, we have detected BuV DNA in the feces of children with severe diarrhea in Bhutan. Additional work is needed to determine this is indeed a human causal pathogen of diarrhea or of any illness. Identification of BuV3 provides evidence of a more diverse population of BuVs than previously documented. Whether this novel genotype is circulating globally or only in Bhutan requires further investigation.
